# Feeding Broiler Chickens with Grape Seed and Skin Meals to Enhance α- and γ-Tocopherol Content and Meat Oxidative Stability

**DOI:** 10.3390/antiox10050699

**Published:** 2021-04-28

**Authors:** Carlos Romero, Maria Nardoia, Ignacio Arija, Agustín Viveros, Ana I. Rey, Marin Prodanov, Susana Chamorro

**Affiliations:** 1 Universidad Católica Santa Teresa de Jesús de Ávila (UCAV), Calle Canteros s/n, 05005 Ávila, Spain; carlos.romero@ucavila.es; 2 Department of Agricultural, Environmental and Food Sciences, University of Molise, 86100 Campobasso, Italy; maria.nardoia@studenti.unimol.it; 3 Department of Animal Production, Faculty of Veterinary Medicine, Universidad Complutense de Madrid, 28040 Madrid, Spain; arijai@ucm.es (I.A.); viverosa@ucm.es (A.V.); anarey@ucm.es (A.I.R.); 4 Department of Production and Characterization of Novel Foods, Institute of Food Science Research (CIAL, CSIC-UAM), C/Nicolas Cabrera 9, Campus de Cantoblanco, Universidad Autonoma de Madrid, 28049 Madrid, Spain; marin.prodanov@uam.es; 5 Department of Genetics, Physiology and Microbiology (Animal Physiology Unit), Faculty of Biology, Complutense University of Madrid, 28040 Madrid, Spain

**Keywords:** polyphenols, grape seed, grape skin, digestibility, tocopherols, antioxidant activity, chickens

## Abstract

Grape seeds (GS) and grape skins (GK) are natural sources of polyphenols with featured antioxidant capacity. An experiment was conducted to investigate the effect of these polyphenol sources in diets formulated to contain the same total extractable grape polyphenol content on growth performance, protein and extractable polyphenol digestibility, plasma and meat α- and γ-tocopherol and thigh meat oxidation in broiler chickens. Five experimental diets were formulated: control, control + vitamin E (200 mg/kg), 30 g/kg GS diet, 110 g/kg GK diet, GS + GK diet (a mixture of 24.4 g/kg GS and 13.1 g/kg GK designed to simulate a reconstituted grape pomace). Feeding chickens with 110 g/kg GK reduced (*p* < 0.001) daily weight gain, worsened (*p* < 0.001) feed conversion ratio, increased (*p <* 0.001) non-extractable polyphenol content in the ileum and in the excreta and decreased (*p <* 0.05) ileal protein digestibility. Regardless of the grape polyphenol source used, the inclusion of grape byproducts in the diets led to an increase of total extractable polyphenol contents in the ileum (*p* < 0.01) and the excreta (*p* < 0.001), which resulted (*p* < 0.001) in a decrease of extractable polyphenol digestibilities. Alpha- and gamma-tocopherol concentrations increased (*p* < 0.001) in plasma and in seven-day stored meat in birds fed the diet combining GS and GK with respect to the control group. As it happened with the vitamin E supplementation, feeding the combination of GS and GK also reduced (*p* < 0.001) the concentration of the lipid peroxidation marker (malondialdehyde) in the stored meat of chickens.

## 1. Introduction

Grape (*Vitis vinifera* L.) is one of the largest fruit crops in the world, with an approximate annual production of 77 million tons [1]. The biggest part of them is destined to the wine production industry. Around 20% of the total weight of grapes used for winemaking results in grape pomace [2], a solid residue left after juice extraction that consists of seeds, skins and stems. Investigations have stressed the importance of byproducts from wine processing as plant materials particularly rich in a wide variety of polyphenols [3,4]. Grape seeds (GS) and skins (GK) are good sources of flavonoids, mainly catechins and procyanidins with diverse degree of polymerization [5,6,7]. Studies have shown that grape polyphenols have the capacity to act as powerful antioxidants by scavenging free radicals and terminating oxidative reactions [8]. The antioxidant activity of grape polyphenols has been reported to improve the oxidative stability in a variety of raw meats: beef [9], chicken [10,11,12], pork [13], lamb [14] and fish [15].

Animal nutrition is currently evolving towards dietary supplementation with unsaturated fatty acids in order to enhance animal fat healthfulness, but this nutritional strategy has been associated with a greater lipoperoxidation in subcutaneous and intramuscular lipids [16]. The negative consequences of lipid oxidation can be overcome by including antioxidants in the diet, such as α-tocopherol (vitamin E). Dietary requirement of this vitamin, which is the antioxidant most commonly included in animal diets, increases thus with the unsaturation degree of dietary fatty acids [17]. Several studies have shown the ability of polyphenols to spare/regenerate tocopherols in rats and pigs [18,19]. In the particular case of broiler chickens, an increase of α-tocopherol content in liver and plasma [20,21,22] has also been reported after feeding diets that included grape pomace and grape seed extracts. The antioxidant potential of grape polyphenols has actually been demonstrated in poultry in several works where a greater meat oxidative stability was detected in chickens fed diets containing different concentrations of grape pomace [10,11,20,23]. Although the positive effect of feeding grape polyphenols on plasma tocopherol content is consistent among studies, its effect on meat tocopherol accumulation seems to be less clear [22]. The effectiveness of grape byproducts depends not only on the total polyphenol content but also on the nature of the polyphenolic compounds incorporated to the diet. In this sense, different antioxidant potential has been observed after feeding broilers with grape skins and grape seeds [11,24]. In general, grape seeds contain higher amounts of phenolic compounds than grape skins. Grape seed polyphenols are constituted only on the base of flavan-3-ol monomers, (+)-catechin, (-)-epicatechin and (-)-epicatechin gallate, whereas those of grape skins contain also (-)-epigallocatechin [25]. Furthermore, seeds are composed of B-type procyanidins with a degree of polymerization up to 17, while grape skins contain both procyanidins and prodelphinidins with a higher degree of polymerization (up to 74) [5,26]. The differences in the nature of polyphenolic compounds and specially, factors such as the degree of polymerization and galloylation, might affect differently the intestinal utilization of grape polyphenols and therefore, their biological activity [27].

Although grape skins and seeds present different antioxidant potential, there are no studies in broiler chickens comparing the biological effects of these two principal components of grape pomace, when separately included in the diet. Thus, the objective of the current study was to evaluate the effect of adding GS, GK and a mixture of these two components, aiming to simulate a reconstituted grape pomace, to diets formulated to contain the same amount of total extractable grape polyphenols on growth performance, protein and extractable polyphenols digestibility, blood and meat α- and γ-tocopherol concentrations and oxidative stability of meat in broiler chickens.

## 2. Materials and Methods

### 2.1. Grape Byproducts

Semi-defatted grape seed (GS) and grape skin (GK) meals (*Vitis vinifera* var. Cencibel) were obtained from red grape pomace at Explotaciones Hermanos Delgado organic winery (Socuéllamos, Ciudad Real, Spain) after 15 days of fermentation. The pomace was dried immediately after pressing in a rotary trommel type dryer with indirectly heated air at temperatures below 80 °C. Seeds and skins were mechanically separated by sieving and air aspiration. Dry skins were ground to particles as big as 1.5 mm. Seeds were subjected to an expeller oil extraction process before grinding.

Proximate composition of semi-defatted GS meal and that of GK meal are shown in Table 1 and Table 2. The α-tocopheryl acetate used in the diets was provided by DSM Nutritional Products Iberia S.A. (Alcalá de Henares, Madrid, Spain).

### 2.2. Solvents and Reagents

All solvents used for HPLC analysis were of liquid chromatography grade and the water was ultrapure. From Sigma-Aldrich (St. Louis, MO, USA), the following products were obtained: Folin–Ciocalteu reagent, α-tocopherol, γ-tocopherol, trolox, butylated hydroxytoluene, 1,1,3,3-tetraethoxy propane and cinnamtannin A2, while acetone, butanol, isopropanol, hexane, acetonitrile and methanol were obtained from Panreac (Castellar del Vallés, Barcelona, Spain). Finally, the following standard compounds were purchased at Extrasynthese (Genay, France): gallic acid, catechin, epicatechin, epicatechin gallate, and procyanidins B1, B2, B3 and C1.

### 2.3. Birds and Diets

A total of 150 one-day-old male broiler Cobb chickens (provided by Avícola Grau, Toledo, Spain) were obtained from a commercial hatchery. The birds were housed in electrically-heated starter battery brooders in an environmentally controlled room with 23 h of constant overhead fluorescent lighting for three weeks. Chickens were allocated to 25 pens, each pen containing six chickens, to receive five dietary treatments during 21 days with five replicates per treatment. Diets in mash form and water were provided *ad libitum*. Ingredients and nutrient composition of diets are shown in Table 3. Celite (Celite Corp., Lompoc, CA, USA), a source of acid-insoluble ash (AIA), was added at 10 g/kg to all diets as an indigestible marker. All diets were formulated to contain similar level of metabolizable energy, crude protein and crude fibre and to meet or exceed the minimum requirements [28] for broiler chickens. Experimental procedures were approved by the University Complutense of Madrid Animal Care and Ethics Committee (protocol code CEA-UCM 20/2012) in compliance with the guidelines for the Care and Use of Animals for Scientific Purposes of the Ministry of Agriculture, Fishery and Food. Experimental diets were as follows: (1) control corn-soybean diet; (2) control + vitamin E (200 mg/kg); (3) GS diet including 30 g/kg of GS meal; (4) GK diet including 110 g/kg of GK meal; (5) GS + GK diet containing 24.4 g/kg of GS meal and 13.1 g/kg of GK meal. Although including different sources of grape polyphenols, diets 3, 4 and 5 were formulated to contain the same amount of total extractable grape polyphenols. The mixture of GS and GK in diet 5 intended to simulate as though a reconstituted grape pomace was being included in the diet. At the end of the experimental period, birds were weighed, and feed consumption was recorded for feed efficiency computation.

### 2.4. Collection of Samples and Measurements

At 19 days of age, clean stainless steel collection trays were placed under each cage, and excreta from the birds were collected for 48 h. A subsample of excreta was collected in polyethylene bags and freeze-dried (Telstar, Terrasa, Spain) for subsequent determination of the contents of celite and extractable and non-extractable polyphenols. At 21 days of age, 15 birds were randomly selected from each treatment (three birds of each replicate of the treatments). Blood was obtained from these birds by cardiac puncture and plasma was prepared for subsequent determination of α-tocopherol and γ-tocopherol. The blood was collected in ethylenediaminetetraacetic (EDTA) vacutainer tubes on ice. Tubes were centrifuged at 2500× *g* for 15 min at 4 °C and the supernatant was removed. All samples were stored at −80 °C until assayed. Thereafter, these fifteen birds per treatment were euthanized with carbon dioxide (100%), the spleen was excised, the ileum was quickly dissected out and the ileal content removed by gentle manipulation into a plastic container and stored at −20 °C. Digesta were pooled from the three birds of each replicate within the same treatment. Ileal contents were freeze-dried and ground (1 mm screen) and used to determine the contents of celite, crude protein and extractable and non-extractable polyphenols. Carcasses from fifty birds (10 birds per treatment) were also immediately trimmed for thigh meat and tissues were individually sampled and used to determine lipid oxidation and α- and γ-tocopherol contents (five replicates of two birds for each treatment). Thigh tissue samples were wrapped in transparent oxygen-permeable polyvinyl chloride film (13,500 cm^3^/m^2^/day), frozen and stored at −20 °C until required. After thawing, the raw meat samples were placed and stored in a nonilluminated refrigerated cabinet at 4 °C. The progress of lipid oxidation and the α- and γ-tocopherol contents were determined in meat samples after 1 and 7 days of refrigerated storage.

### 2.5. Chemical Analyses

Dry matter (930.15), crude protein (976.05), crude fibre (978.10) and gross energy were analysed according to the methods of the AOAC [29]. Neutral detergent fibre (NDF), acid-detergent fibre (ADF) and acid-detergent lignin (ADL) were determined according to the sequential method of Van Soest et al. [30]. Ether extract was determined by extraction in petroleum ether after acidification with 4 N HCl solutions [31]. The AIA contents of diet, ileal content and excreta were measured after ashing the samples and treating the ashes with boiling 4 M HCl [32].

#### 2.5.1. Extractable and Non-Extractable Polyphenol Contents

For the extraction of phenolic compounds, 0.50 g of sample was placed in a capped centrifuge tube, suspended in 20 mL of acidic methanol/water (50:50 *v*/*v*, pH = 2) and thoroughly shaken at room temperature for 1 h. The tube was centrifuged at 3500 rpm for 15 min and the supernatant was separated. Twenty millilitres of acetone/water (70:30 *v*/*v*) were added to the residue and shaking and centrifugation were repeated. The methanol and acetone extracts were combined and used for total extractable polyphenol characterization and quantification. The residue remaining after extraction was dried and used to determine the non-extractable polyphenol (NEP) content. Total extractable polyphenol (TEP) content in GS and GK meals, experimental diets, ileal digesta and excreta were determined by the Folin–Ciocalteu procedure [33]. Briefly, a mixture of 0.5 mL of extract, 0.5 mL of the Folin–Ciocalteu reagent (Sigma-Aldrich, St. Louis, MO, USA) and 10 mL of 1 M Na_2_CO_3_ were introduced in a 25 mL volumetric flask. After reacting for 1 h, absorbance was measured at 750 nm using an ultraviolet-visible spectrophotometer (Hitachi U-2000; Hitachi, Ltd., Tokyo, Japan). Absorbance values were compared against a standard curve made with gallic acid (Sigma-Aldrich, St. Louis, MO, USA) ranging from 0.05 to 0.5 mg of gallic acid/mL. Results were expressed as grams of gallic acid equivalents (GAE) per 100 g of DM.

The identification and quantification of the different extractable phenolic compounds present in GS and GK were performed by HPLC-QTOF-MS consisting in a HPLC (Agilent 1200, Agilent Technologies, Santa Clara, CA, USA) coupled with a diode array detector (Agilent G1315B) and an Accurate-Mass Quadrupole Time of Flight (QTOF) detector (Agilent 6530) with ESI-Jet Stream Technology (Agilent Technologies, Waldbroon, Germany). Separation was performed on a Zorbax Eclipse Plus C18 100 mm × 3.5 µm × 4.6 mm column (Agilent) with a pre-column (Sigma-Aldrich, St. Louis, MO, USA). A mobile phase composed of solvents A (water:formic acid, 99.9:0.1, *v*/*v*) and B (acetonitrile:formic acid, 99.9:0.1, *v*/*v*) was applied at a flow rate of 0.5 mL/min. Solvent gradient was as follows: 10% B at 0 min, 30% B at 15 min, 30% B at 30 min, 80% B at 32 min, 10% B at 35 min and 10% at 45 min. The electrospray ionization (ESI) parameters were as follows: drying gas flow, 8 L/min; nebulizer pressure, 45 psi; gas drying temperature, 350 °C; sheath gas temperature, 325 °C; sheath gas flow, 11 L/min; capillary tension, 3500 kV; nozzle tension, 500 V; and fragmentator, 100 V. The ESI was operated in negative mode. Data were collected in extended dynamic range, 100–1000 *m*/*z*. Data acquisition and processing were carried out with the Masshunter Data Acquisition B.05.01 and Masshunter Qualitative Analysis B.07.00 SP2 software. Compounds were identified by comparing mass spectra with the corresponding standard if available and confirmed by comparison with the retention time of the standard. In the case of compounds with standards that were not available, identification was based on prediction of chemical formula from accurate ion mass measurement and quantification was performed by interpolation into the calibration curves of some structurally related compounds: procyanidin gallates with procyanidin B2; procyanidin trimers with procyanidin C1 and procyanidin tetramer with cinnamtannin A2.

The acid butanol assay [34] was used to quantify the total non-extractable polyphenols remaining in the residue of GS and GK meals and in that of the diets after the extraction of phenolic compounds like previously described. A stock solution of 0.07% (*w*/*v*) FeSO_4_.7H_2_O dissolved in 95:5 (*v*/*v*) 1-butanol/HCl was prepared. In a test tube, 7 mL of the stock solution and 50 mg of sample were mixed and heated for 50 min at 95 °C. The mixtures were cooled in an ice bath and after centrifugation the absorbance was measured at 550 nm using an ultraviolet-visible spectrophotometer Hitachi U-2000 (Hitachi, Ltd.). The non-extractable polyphenol content was expressed as cyanidin equivalent after plotting a standard curve of cyanidin ranging from 0 to 333 mg/L (*R^2^* = 0.999).

#### 2.5.2. Plasma and Meat α-Tocopherol and γ-Tocopherol Assessment

The concentrations of α- and γ-tocopherol in plasma and in meat stored under refrigeration for 1 and 7 days were determined following the method of Buttriss and Diplock [35], which includes saponification with saturated KOH in the presence of pyrogallol. Briefly, 400 µL of plasma or 100 mg of freeze-dried thigh meat were extracted with hexane in three phases, evaporated and diluted with 1 mL of hexane. The α- and γ-tocopherol contents were measured by normal-phase HPLC using a Hypersil Si 100 (5 µm) column and a mobile phase of hexane-isopropanol (98:2 *v*/*v*).

#### 2.5.3. Meat Lipid Oxidation

The extent of lipid oxidation in meat was determined by measuring the thiobarbituric acid reactive substances (TBARS) after 1 and 7 days of refrigerated storage of meat using the procedure described by Botsoglou et al. [36]. Five grams of ground meat were homogenized with 10 mL of 5% trichloroacetic acid in an Ultraturrax at 21,280× *g* for 1 min. Butylated hydroxytoluene was added prior to homogenization at a level of 125 μg/mg of fat. The blended sample was filtered through Whatman number 2V filter (Whatman International Ltd., Maidstone, UK) and 2.5 mL of the filtrate were mixed with 1.5 mL of 0.8% thiobarbituric acid in distilled water in capped test tubes. Tubes were vortexed, incubated at 70 °C for 30 min and absorbance was determined at 532 nm using an ultraviolet-visible spectrophotometer Hitachi U-2000 (Hitachi, Ltd.). Results were expressed as mg of malondialdehyde (MDA) per kilogram of muscle after the preparation of a standard curve of 1,1,3,3-tetraethoxy propane.

### 2.6. Calculations and Statistical Analysis

Ileal digestibility (ID) of CP and TEP was determined by using the AIA content and calculated with the following formula:

ID = 100% − [100% × (AIA concentration in feed/AIA concentration in ileal content or excreta) × (CP or TEP concentration in ileal content or excreta/CP or TEP concentration in feed)].

Data were subjected to a one-way analysis of variance (ANOVA) by using the general linear model procedure (Version 9.4, SAS Institute Inc., Cary, NC, USA). When the effect was declared significant (*p* < 0.05), means were compared using a Duncan’s multiple-range test. Non-orthogonal contrasts were used to test differences between the combined means of several groups. The pen of six chickens was regarded as the experimental unit for the measurement of the following parameters: growth performance and ileal and excreta contents and digestibilities. However, the individual bird was considered the experimental unit in the determination of spleen weight, blood and meat α- and γ-tocopherol concentrations and TBARS content.

## 3. Results

### 3.1. Growth Performance

Growth performance of broiler chickens is reported in Table 4. The dietary inclusion of GK at 110 g/kg reduced (*p* < 0.001) daily weight gain by 18.2% and increased (*p* < 0.001) feed conversion ratio by 15.6% as compared with the control diet. No effect of dietary treatments was observed on daily feed intake (49.9 g/d, on average).

No significant differences (*p* > 0.05) were found among dietary treatments on spleen weight (0.074% of body weight, on average).

### 3.2. Ileal and Excreta Extractable and Non-Extractable Polyphenol Contents

Ileal and excreta TEP and NEP contents are reported in Table 5. As expected, ileal and excreta TEP and NEP contents were not affected by the inclusion of vitamin E in the diet, as compared with the control group. The dietary inclusion of the grape byproducts increased TEP content both in the ileum (by 21.7%, *p* < 0.01) and in the excreta (by 34.4%, *p* < 0.001). As regards NEP, birds fed the diet containing 110 g/kg of GK were the ones showing the highest content in the ileum (187% higher than in the control birds, *p* < 0.001) and in the excreta (417% higher than in the control birds, *p* < 0.001).

### 3.3. Protein and Polyphenol Digestibility

**Figure 1:**
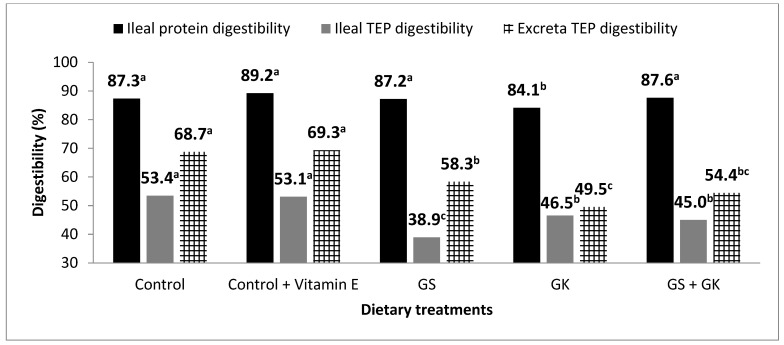
Ileal digestibility of protein (SEM^1^ = 0.535, *) and ileal (SEM = 1.37, ***) and excreta (SEM = 2.16, ***) digestibility of total extractable polyphenols (TEP) in broiler chickens fed diets containing grape seed (GS) meal, grape skin (GK) meal or vitamin E. Different letters for the same parameter (^a, b, c^) indicate significant differences (*p* < 0.05). ^1^ SEM, standard error of means; each value represents the mean of five replicates (three birds per replicate for ileum and six birds per replicate for excreta). * *p* < 0.05, *** *p* < 0.001.

Ileal protein digestibility and ileal and excreta TEP digestibility are reported in Figure 1. Ileal protein digestibility was reduced by 4.75% (*p* = 0.024) with the dietary inclusion of 110 g/kg of GK. Feeding grape byproducts led to lower ileal (18.6% lower, *p* < 0.001) and excreta (21.3% lower, *p* < 0.001) TEP digestibilities as compared with the control group. Among the different dietary treatments including grape byproducts, the lowest ileal TEP digestibility was obtained with the diet including GS at 30 g/kg (38.9 vs. 45.7% for the rest of the grape byproducts treatments, *p* < 0.01). As concerns excreta TEP digestibility, the dietary inclusion of GK, either individually or combined with GS, reduced TEP digestibility by 10.9% (*p* < 0.05).

### 3.4. Plasma α- and γ-Tocopherol Concentrations

The effect of dietary treatments on plasma α- and γ-tocopherol concentrations is reported in Table 6. Birds supplemented with vitamin E in the diet showed the highest α-tocopherol concentration in plasma, as compared with the rest of the dietary treatments (36.1 vs. 5.92 μg/mL, *p* < 0.001). However, the plasma α-tocopherol concentration was also higher in birds fed the diets containing the grape byproducts than in those fed the control diet (6.62 vs. 3.84 μg/mL, *p* < 0.01). Among the dietary treatments including grape byproducts, the diet combining GS and GK was the one that led to the highest plasma α-tocopherol concentration (142% higher than in the control birds, *p* < 0.001). In fact, the latter diet was the only one that increased plasma γ-tocopherol concentration, as compared with the rest of the dietary treatments (0.867 vs. 0.565 μg/mL, *p* < 0.001).

### 3.5. Meat α- and γ-Tocopherol Concentrations and Lipid Oxidation

The concentrations of α- and γ-tocopherol in chicken meat, as well as the extent of meat lipid oxidation, measured as malondialdehyde (MDA), after 1 and 7 days of meat storage, are shown in Table 7. On day 1 of storage, the highest α-tocopherol concentration in thigh meat was obtained in birds fed the diet supplemented with vitamin E, as compared with the rest of the dietary treatments (71.1 vs. 8.71 μg/g, *p* < 0.001). After seven days of refrigerated storage, still the highest concentration of α-tocopherol in meat was observed in birds receiving the diet enriched with vitamin E (10.6 vs. 2.79 μg/g, *p* < 0.001). Nonetheless, on day 7 of storage it was also detected that meat α-tocopherol concentration was higher in birds fed the diet combining GS and GK than in those fed the control diet (4.56 vs. 1.64 μg/g, *p* < 0.001). As regards γ-tocopherol concentration in thigh meat, birds fed the diet supplemented with vitamin E were those showing on day 1 the highest meat γ-tocopherol concentration (4.02 vs. 2.12 μg/g, *p* < 0.001), whereas on day 7 of storage the highest γ-tocopherol concentration in thigh meat was observed in birds fed the diet combining GS and GK (0.810 vs. 0.421 μg/g, *p* < 0.01).

On day 1 of storage, no significant differences among the dietary treatments were detected for the MDA amount in broilers’ meat (0.275 mg MDA/kg meat, on average). However, after seven days of refrigerated storage of thigh meat, the MDA concentration was lower (*p* < 0.001) in chickens supplemented with vitamin E and in those fed the combination of GS and GK than in the chickens receiving the other diets. Besides, on day 7 of storage, no significant difference on MDA concentration was found between the supplementation with vitamin E and the dietary inclusion of both GS and GK. Furthermore, for the birds fed the diet combining GS and GK, values for meat MDA concentration did not differ significantly (*p* > 0.05) after seven days of storage with respect to the values recorded on day 1. In fact, the increase of MDA concentration after seven days of meat storage was much lower (*p* < 0.001) in birds fed the GS&GK diet (126%, on average) than in those birds fed the control, GS and GK diets (523%, on average).

## 4. Discussion

### 4.1. Chemical Composition and Polyphenolic Content of Grape Seed and Skin Meals

The chemical composition and the main extractable phenolic compounds identified in grape seed and skin meals are reported in Table 1 and Table 2, respectively. In the present experiment, grape seed meal presented a higher content of total extractable polyphenols than grape skin meal, which is in agreement with findings previously reported by other authors [7,37,38]. Furthermore, while the grape seed meal contained a higher amount of flavanols, grape skins contained a greater variety of other phenolic compounds, such as phenolic acids. Among the latter, hydroxycinnamoyl tartaric acids (esters of caffeic, p-coumaric and ferulic acids with tartaric acid) are particularly abundant in the berry juice and skins. Anthocyanin compounds, which are responsible for the red colour of grapes, are present in the skins and can also contribute to their antioxidant potential. However, the assessment of anthocyanins was not included in the present study, since their identification requires a mass spectra acquisition in positive mode. Concerning the non-extractable polyphenol fraction, which includes those polyphenols that remain bound to the insoluble solid substrate after conventional solvent extraction, a similar content was observed for grape seeds and grape skins in the present study. This fraction comprises mainly proanthocyanidins, phenolic acids and hydrolysable tannins that are closely linked with the food matrix [39], and might differ among grape seeds and skins. In this sense, grape skin procyanidins present a higher degree of polymerization than grape seed procyanidins [5,26].

As aforementioned, in the current study the diets including grape byproducts were formulated to contain the same amount of total extractable grape polyphenols (2.4 g/kg). This dietary dose of grape polyphenols was chosen as in previous research [10,21] a dietary concentration of 2.34 g/kg of grape polyphenols demonstrated to be effective in increasing tocopherol concentration and in enhancing meat oxidative stability without impairing chickens growth performance. Taking into account the different polyphenolic profile of grape seeds and skins, differences among the dietary treatments of the study should actually be ascribed to the different kind of phenolic compounds contained in the diets, and not to the amount of total extractable polyphenols. Furthermore, dietary treatments also differed in the non-extractable polyphenol concentration, as shown in Table 3.

### 4.2. Growth Performance and Protein and Polyphenol Intestinal Utilization

Albeit having the same dietary concentration of grape polyphenols (2.4 g/kg, on average), the different grape byproduct treatments of this study did not have the same effect on the growth performance of chickens. While no effect on productive performance was observed with the diet containing solely GS and with the diet containing the combination of GS and GK, the diet including GK at 110 g/kg impaired chickens growth performance, as this dietary treatment resulted in a reduction of daily weight gain and in an increase of feed conversion ratio.

Previous experiments have shown that the effect of grape polyphenols on chicken growth performance depends both on their dietary dose and on the polyphenol source. With a dietary concentration of total extractable grape polyphenols (2.34 g/kg) similar to that of the grape byproduct diets of the present study, a previous dietary inclusion of grape pomace at 10% [10] had not caused any negative effect on chicken performance. Nevertheless, in another study [24] a higher dietary concentration of grape polyphenols (4.27 g/kg), obtained with an inclusion of unfermented grape skins at 6%, impaired both chicken growth rate and feed conversion ratio. Regarding grape seed extracts, a detrimental effect on growth performance was observed from a dietary grape polyphenol dose of 1.48 g/kg [40,41]. Given that the dietary treatments in the present study were formulated to be isonutritional and also to contain a similar amount of total extractable grape polyphenols, the different effects observed for the grape byproduct-based diets could be attributed to the different kinds of polyphenols contained in GS and GK (Table 2) and to the different concentration of non-extractable polyphenols, which was higher for GK diet than for GS or GS&GK diets. Grape skins differ from grape seeds primarily by containing a lower amount of proanthocyanidins, but also because of the higher degree of polymerization of these procyanidins [37,38]. In fact, an appreciable amount of the polyphenols present in the food matrix is linked to polymeric molecules like dietary fibre and/or proteins and remains in the corresponding insoluble residues after the extraction. These are the so-called non-extractable polyphenols [39]. In the present study, a similar content of this non-extractable fraction was observed in GS and GK but then the addition of 3% GS and of 11% GK led to a higher dietary content of these non-extractable polyphenols in the GK diet. The characterization of these non-extractable polyphenols has not been included in the present study but it could have been useful to explain the different responses obtained with GS and GK treatments. Furthermore, studies dealing with the quantification and characterization of this non-extractable phenolic fraction in raw materials are still scarce [39].

Taking into account that the GS and GK meals used in this study had a similar content of non-extractable polyphenols, the fact of including 30 g/kg of GS and 110 g/kg of GK in the diets made the GK diet present a concentration of non-extractable polyphenols around three to four times higher than that of the other two grape byproduct diets. In fact, the GK diet resulted in the highest ileal concentration of non-extractable polyphenols and was the only dietary treatment causing a reduction of the ileal digestibility of protein. All in all, it can be surmised that the presence of a high amount of non-extractable polyphenols in the ileum could have hindered the intestinal digestion of proteins. Polyphenols are known to form complexes with proteins due to the interaction of their reactive hydroxyl groups with the carbonyl groups of proteins. This is particularly true in the case of proanthocyanidins that stand out among the different phenolic compounds for their high polarity [42]. In this sense, non-extractable polyphenols have traditionally been deemed antinutritional factors since the consumption of ingredients rich in tannins negatively affects feed palatability and hence feed intake, nutrient utilization in the intestine and thereby animal performance. For instance, several publications [43,44] have highlighted the detrimental effect of a high dietary inclusion of tannin-rich ingredients such as sorghum and faba bean in poultry diets. In previous research works conducted by our team in which the dietary inclusion of GK [24] or that of other grape byproducts like grape seed extract [40] or grape pomace [21] made the diets contain a high concentration of non-extractable polyphenols (from 0.740 to 2.28 g/kg), ileal digestibility of protein of broiler chickens was decreased in a similar extent (from 2.37 to 6.97%) to that observed in the current work (4.75%).

Ileal content of TEP was increased with the inclusion of grape byproducts in the diet. As a result, ileal TEP digestibility was lower for chickens fed the grape byproducts diets than for the control birds. In the excreta, TEP content was also higher for birds fed grape byproducts. These results agree with previous findings also reporting the effect of feeding grape pomace, grape seed extract or grape skin on grape polyphenol digestibility [10,21,23,24,45]. Additionally, in the present study we also observed differences in the digestibility of polyphenols when comparing GS and GK treatments. Specifically, birds fed GS diet showed a lower ileal TEP polyphenol digestibility and a higher excreta TEP digestibility than birds fed GK diet. Birds fed the diet combining GS and GK meals presented intermediate values. The different response observed among dietary treatments could also be caused by the different polyphenol composition present in seeds and skins [46]. Monomeric and some oligomeric polyphenols are thought to be directly absorbed at the small intestine [47], while polymeric forms cannot be absorbed in their native forms in the ileum and are hydrolysed by gut microbiota in the ceca, giving rise to small phenolic acids that are likely to have better bioavailability [48,49]. The extensive intestinal utilization and microbial metabolism of grape polyphenols have also been recently demonstrated in poultry [27]. However, factors such as a higher degree of polymerization and gallic acid esterification can reduce the intestinal utilization of grape catechins in poultry [27] and consequently, might contribute to explain the differences found among treatments of the present work.

### 4.3. Plasma and Meat α- and γ-Tocopherol Concentrations and MDA Values

The combined inclusion in the diet of GS and GK, which resulted in a dietary grape polyphenol content of 2.27 g/kg, increased the concentrations of both α- and γ-tocopherol in plasma as well as in 7-day stored raw meat. It should be noted that the higher concentrations of α- and γ-tocopherol were reached both in plasma and in stored meat with the dietary combination of GS and GK, instead of including these grape byproducts individually in the diet. In previous research also conducted with broiler chickens, the inclusion in the diet of grape pomace increased the concentration of α-tocopherol in plasma (with 1.17 g/kg of grape polyphenols in the diet, content obtained by including 5% of GP [21]) and in raw thigh meat (with 2.34 g/kg of grape polyphenols in the diet, by including 10% of GP [10]). As aforementioned, in the present experiment the best results concerning α-tocopherol and γ-tocopherol concentrations were achieved with the combined inclusion of GS and GK in the diet, designed to meet the composition of GP. Furthermore, in a recent study [22], the dietary inclusion of grape pomace also increased the concentration of γ-tocopherol in the thigh meat of broiler chickens, whereas the individual inclusion of grape seed extract in the diet of chickens did not result in a significantly higher meat γ-tocopherol concentration. This higher content of tocopherols with the combined inclusion of GS and GK could be attributed to their protective effect between them or to a greater variety of polyphenolic compounds.

In keeping with the results found for meat α-tocopherol concentration, both the dietary supplementation with vitamin E and the combined inclusion of GS and GK in the diet managed to retard lipid oxidation in the thigh meat of chickens after seven days of refrigerated storage of meat. Besides, no significant difference arose between these two dietary treatments for MDA values. These results are consistent with previous findings of our laboratory [10,23,24] since dietary doses of grape polyphenols ranging from 1.17 to 2.92 g/kg also reduced MDA concentration in chicken stored meat, with no significant difference with respect to the dietary supplementation with vitamin E. Interestingly, despite in the present study grape byproducts treatments contained a similar concentration of grape polyphenols, the meat lipid oxidation was only mitigated with the dietary combination of GS and GK, designed to meet grape pomace composition, whereas the individual inclusion of GS or GK in the diet did not reduce MDA values. As reported before, the combination of GS and GK resulted in the highest content of γ-tocopherol when compared to the other groups. Previous investigations have reported the potent effect of the combination of both tocopherols and the important contribution of γ-tocopherol rather than the unique use of α-tocopherol for stabilizing lipid oxidation [50,51].

In addition, the antioxidant effect reached when feeding grape byproducts could be related to the increased concentration of α-tocopherol detected both in plasma and in stored meat. On the one hand, the concentration of α-tocopherol may simply rise because grape byproducts contain moderate levels of this vitamin [52] and may be providing it to the animals through the diet. However, in our experiment, the total concentration of vitamin E added by the dietary inclusion of grape byproducts represents a low proportion of the total vitamin E present in the whole diet, but, on the other hand, a regenerating effect of α-tocopherol could also be occurring due to the intake of grape polyphenols, as it has already been detected in chickens [20,21,22]. Alpha-tocopherol has the ability to trap the lipid peroxide radical and during this process another radical (α-tocopheroxyl) is generated. This new radical should be reduced by the action of other coexisting hydrophilic antioxidants (such as vitamin C or polyphenols) in order to newly regenerate α-tocopherol. The role of polyphenols in this process has been demonstrated by Iglesias et al. [53], who showed the capacity of grape procyanidins to recycle the oxidized α-tocopherol. Likewise, also Simonetti et al. [54] suggested that dietary procyanidins from grape exert their in vivo antioxidant protection by sparing liposoluble vitamin E. This sparing and regenerating effect of α-tocopherol has also been detected with polyphenols originating from other sources in rat, human, cattle and pig models [18,19,55].

## 5. Conclusions

Our results showed that the antioxidant potential of dietary grape polyphenols in chickens is affected by the source of grape polyphenols. Thus, although with the same dietary content of grape extractable polyphenols, the dietary combination of GS and GK led to better results than the separate dietary inclusion of these grape byproducts. In fact, the dietary treatment including both GS and GK resulted in higher γ-tocopherol concentrations, both in plasma and in stored meat, and in a lower meat lipid oxidation than the diets including separately GS or GK. On the other hand, the sole inclusion of GK in the diet impaired growth performance and ileal protein digestibility of chickens. Since the grape byproduct diets of this study presented a similar amount of total extractable grape polyphenols, differences detected among the dietary treatments could be attributed to the different profile of the phenolic compounds contained in GS and GK. Hence, future studies should assess the optimal proportions of GS and GK in the diet of broiler chickens in order to maximise antioxidant activity without reducing growth rate.

## Figures and Tables

**Table 1:** Proximate composition (% dry matter) of grape seed (GS) and grape skin (GK) meals.

Nutrients	g/100 g ^1^	
	GS Meal	GK Meal
Crude protein (CP)	19.6 ± 0.41	16.3 ± 0.00
Crude fibre	25.4 ± 1.87	14.5 ± 1.33
Neutral detergent fibre (NDF)	52.9 ± 2.36	49.7 ± 0.85
Acid detergent fibre (ADF)	44.6 ± 2.10	41.2 ± 1.08
Acid detergent lignin (ADL)	38.0 ± 1.83	28.3 ± 1.40
Ether extract	7.17 ± 0.31	7.06 ± 0.33
Total extractable polyphenols	7.93 ± 0.60	2.35 ± 0.14
Non-extractable polyphenols	1.15 ± 0.20	1.24 ± 0.10
Gross energy (cal/g)	5062 ± 13.6	4500 ± 19.8

^1^ Data are the mean of four determinations ± SD.

**Table 2:** Main extractable phenolic compounds identified in grape seed (GS) and grape skin (GK) meals expressed as mg per 100 g of DM (dry matter).

Phenolic Compounds		GS Meal	GK Meal
Flavanol monomers	Catechin	108.0 ± 5.1	11.6 ± 0.60
	Epicatechin	93.5 ± 7.0	9.68 ± 0.73
	Epicatechin 3-*O*-gallate	5.21 ± 0.10	0.71 ± 0.01
Flavanol dimers	Procyanidin B1	32.0 ± 1.11	3.81 ± 0.14
	Procyanidin B2	32.9 ± 1.14	4.26 ± 0.16
	Procyanidin B3	30.2 ± 1.82	1.51 ± 0.29
	Procyanidin gallate 1 ^1^	8.72 ± 0.84	3.04 ± 0.15
	Procyanidin gallate 2 ^1^	29.4 ± 3.13	4.67 ± 0.32
Flavanol trimers	Procyanidin C1	19.7 ± 1.52	2.92 ± 0.23
	Procyanidin trimer 2 ^2^	6.99 ± 0.52	1.16 ± 0.09
	Procyanidin trimer 3 ^2^	16.7 ± 1.61	2.29 ± 0.23
	Procyanidin trimer 4 ^2^	8.43 ± 0.58	1.25 ± 0.09
Flavanol tetramers	Procyanidin cinnamtannin A2	25.7 ± 0.69	5.20 ± 0.14
	Procyanidin tetramer ^3^	9.78 ± 0.10	3.55 ± 0.20
Phenolic acids	Gallic acid	72.2 ± 5.60	39.1 ± 3.80
	Caftaric acid	nd ^4^	25.2 ± 2.50
	Fertaric acid	nd	14.1 ± 0.50
	Coutaric acid	nd	9.78 ± 0.30
Total extractable polyphenols, g GAE ^5^/100 g DM		7.93 ± 0.60	2.35 ± 0.14

^1^ Compounds identified by prediction of chemical formula from accurate ion mass measurement. Quantified by using the calibration curve of procyanidin B2. ^2^ Compounds identified by prediction of chemical formula from accurate ion mass measurement. Quantified by using the calibration curve of procyanidin C1. ^3^ Compounds identified by prediction of chemical formula from accurate ion mass measurement. Quantified by using the calibration curve of cinnamtannin A2. ^4^ nd: not detected. ^5^ GAE: gallic acid equivalents.

**Table 3:** Ingredients and nutrient composition of experimental diets (g/kg as fed).

Ingredients	Control	Control + Vit E ^1^	GS ^2^	GK ^3^	GS + GK
Corn (8.1% CP)	410	410	415	364	409.3
Soybean (48% CP)	383	383	373	359	371
Sunflower oil	100	100	100	100	100
Salt	3	3	3	3	3
Monocalcium phosphate	17.8	17.8	17.8	17.8	17.8
Calcium carbonate	14.2	14.2	14.2	14.2	14.2
Vitamin-mineral premix ^4^	5	5	5	5	5
DL-Methionine	2	2	2	2	2.2
Straw	55	55	30	15	30
Grape seed meal	0	0	30	0	24.4
Grape skin meal	0	0	0	110	13.1
Celite ^5^	10	10	10	10	10
**Analysed composition**					
Total extractable polyphenols	1.7	1.7	4.09	4.18	3.97
Non-extractable polyphenols	0.075	0.07	0.324	1.46	0.413
Crude protein	208	207	205	207	209
Ether extract	122	122	124	126	122
Crude fibre	48	48	46.6	45.8	46.5
**Calculated composition**					
AME ^6^ (kcal/kg)	3106	3106	3119	2963	3099
Lysine	12.4	12.4	12.2	12.2	12.2
Met + Cys	8.8	8.8	8.82	8.77	9
Ca	10.5	10.5	10.6	11.1	10.8
Available P	4.5	4.5	4.5	4.5	4.5

^1^ This diet included 200 mg/kg of α-tocopheryl acetate. ^2^ GS = Grape seeds. ^3^ GK = Grape skins. ^4^ Vitamin-mineral mix supplied the following per kilogram of diet: vitamin A, 8250 IU; cholecalciferol, 1000 IU; vitamin E, 11 IU; vitamin K, 1.1 mg; vitamin B_12_, 12.5 μg; riboflavin, 5.5 mg; Ca panthothenate, 11 mg; niacin, 53.3 mg; choline chloride, 1020 mg; folic acid, 0.75 mg; biotin, 0.25 mg; delquin, 125 mg; DL-Met, 500 mg; amprol, 1 g; Mn, 55 mg; Zn, 50 mg; Fe, 80 mg; Cu, 5 mg; Se, 0.1 mg; I, 0.18 mg; and NaCl, 2500 mg. ^5^ Celite Corp, Lompoc, CA. ^6^ AME = apparent metabolisable energy.

**Table 4:** Performance of broiler chickens (1 to 21 days) fed diets containing grape seed (GS) meal, grape skin (GK) meal or vitamin E.

Dietary Treatments	Daily Weight Gain (g/d)	Daily Feed Intake (g/d)	Feed Conversion Ratio
Control	35.8 ^a^	50.4	1.41 ^b^
Control + Vitamin E	38.2 ^a^	52.6	1.38 ^b^
GS	34.3 ^a^	48.6	1.42 ^b^
GK	29.3 ^b^	47.8	1.63 ^a^
GS + GK	35.5 ^a^	49.9	1.41 ^b^
SEM ^1^	1.25	1.86	0.02
*p*-value ^2^	***	ns	***

Different letters in the same column (^a, b^) indicate significant differences (*p* < 0.05). ^1^ SEM, standard error of means; each value represents the mean of five replicates (six birds per replicate). ^2^ ns: no significant effect (*p* > 0.05). *** *p* < 0.001.

**Table 5:** Ileal and excreta total extractable and non-extractable polyphenol contents in broiler chickens fed diets containing grape seed (GS) meal, grape skin (GK) meal or vitamin E.

Dietary Treatments	Total Extractable Polyphenols (g GAE ^1^/100 g)		Non-Extractable Polyphenols (mg Cyanidin/100 g)	
	Ileal	Excreta	Ileal	Excreta
Control	0.467 ^c^	0.372 ^c^	0.032 ^b^	0.023 ^c,d^
Control + Vitamin E	0.457 ^c^	0.336 ^c^	0.032 ^b^	0.016 ^d^
GS	0.624 ^a^	0.461 ^b^	0.060 ^b^	0.045 ^b,c^
GK	0.564 ^a,b^	0.551 ^a^	0.092 ^a^	0.119 ^a^
GS + GK	0.517 ^b,c^	0.488 ^b^	0.047 ^b^	0.072 ^b^
SEM ^2^	0.03	0.015	0.008	0.009
*p*-value ^3^	**	***	***	***

Different letters in the same column (^a, b, c, d^) indicate significant differences (*p* < 0.05). ^1^ GAE: gallic acid equivalents. ^2^ SEM, standard error of means; each value represents the mean of five replicates (three birds per replicate for ileum and six birds per replicate for excreta). ^3^ ** *p* < 0.01 *** *p* < 0.001.

**Table 6:** Effect of dietary inclusion of grape seed (GS) meal, grape skin (GK) meal or vitamin E on blood α- and γ-tocopherol concentration (μg/mL) in 21-day old broiler chickens.

Dietary Treatments	α-Tocopherol	γ-Tocopherol
Control	3.84 ^c^	0.533 ^b^
Control + Vitamin E	36.1 ^a^	0.662 ^b^
GS	4.69 ^b,c^	0.563 ^b^
GK	5.89 ^b,c^	0.501 ^b^
GS + GK	9.28 ^b^	0.867 ^a^
		
SEM ^1^	1.31	0.065
*p*-value^2^	***	**

Different letters in the same column (^a, b, c^) indicate significant differences (*p* < 0.05). ^1^ SEM, standard error of means; each value represents the mean of five replicates (three birds per replicate). ^2^ ** *p* < 0.05 *** *p* < 0.001.

**Table 7:** Effect of refrigerated storage on lipid oxidation (mg MDA/kg) and α and γ tocopherol content (μg/g) in the thigh meat of broiler chickens fed diets containing grape seed (GS) meal, grape skin (GK) meal or vitamin E.

Dietary Treatments	α-Tocopherol		γ-Tocopherol		TBARS ^1^	
	1 d	7 d	1 d	7 d	1 d	7 d
Control	9.89 ^b^	1.64 ^c^	2.24 ^b^	0.414 ^b^	0.288	1.88 ^a^
Control + Vitamin E	71.1 ^a^	10.6 ^a^	4.02 ^a^	0.487 ^b^	0.283	0.870 ^b^
GS	8.25 ^b^	1.46 ^c^	2.11 ^b^	0.336 ^b^	0.282	1.70 ^a^
GK	4.69 ^b^	3.50 ^b,c^	1.97 ^b^	0.449 ^b^	0.267	1.61 ^a^
GS + GK	12.0 ^b^	4.56 ^b^	2.16 ^b^	0.810 ^a^	0.255	0.581 ^b^
SEM ^2^	2.89	0.678	0.224	0.073	0.011	0.171
*p*-value ^3^	***	***	***	**	ns	***

Different letters in the same column (^a, b, c^) indicate significant differences (*p* < 0.05). ^1^ TBARS: thiobarbituric acid reactive ng substances. MDA: malondialdehyde. ^2^ SEM, standard error of means; each value represents the mean of five replicates (two birds per replicate). ^3^ ns: no significant effect (*p* > 0.05) ** *p* < 0.05, *** *p* < 0.001.

## Data Availability

Not applicable.
